# Transcriptomic and Lipidomic Mapping of Macrophages in the Hub of Chronic Beta-Adrenergic-Stimulation Unravels Hypertrophy-, Proliferation-, and Lipid Metabolism-Related Genes as Novel Potential Markers of Early Hypertrophy or Heart Failure

**DOI:** 10.3390/biomedicines10020221

**Published:** 2022-01-20

**Authors:** Sophie Nadaud, Mathilde Flamant, Wilfried Le Goff, Elise Balse, Catherine Pavoine

**Affiliations:** INSERM, Institute of Cardiometabolism and Nutrition (ICAN), Sorbonne Université, UMR_S1166, F-75013 Paris, France; sophie.nadaud@sorbonne-universite.fr (S.N.); flamantmathilde@gmail.com (M.F.); wilfried.le_goff@upmc.fr (W.L.G.); elise.balse@sorbonne-universite.fr (E.B.)

**Keywords:** cardiac hypertrophy and heart failure, sympathetic nervous system and chronic beta-adrenergic signaling, temporal cardiac-resident macrophage plasticity, RNA sequencing and lipidomic analysis

## Abstract

Sympathetic nervous system overdrive with chronic release of catecholamines is the most important neurohormonal mechanism activated to maintain cardiac output in response to heart stress. Beta-adrenergic signaling behaves first as a compensatory pathway improving cardiac contractility and maladaptive remodeling but becomes dysfunctional leading to pathological hypertrophy and heart failure (HF). Cardiac remodeling is a complex inflammatory syndrome where macrophages play a determinant role. This study aimed at characterizing the temporal transcriptomic evolution of cardiac macrophages in mice subjected to beta-adrenergic-stimulation using RNA sequencing. Owing to a comprehensive bibliographic analysis and complementary lipidomic experiments, this study deciphers typical gene profiles in early compensated hypertrophy (ECH) versus late dilated remodeling related to HF. We uncover cardiac hypertrophy- and proliferation-related transcription programs typical of ECH or HF macrophages and identify lipid metabolism-associated and Na^+^ or K^+^ channel-related genes as markers of ECH and HF macrophages, respectively. In addition, our results substantiate the key time-dependent role of inflammatory, metabolic, and functional gene regulation in macrophages during beta-adrenergic dependent remodeling. This study provides important and novel knowledge to better understand the prevalent key role of resident macrophages in response to chronically activated beta-adrenergic signaling, an effective diagnostic and therapeutic target in failing hearts.

## 1. Introduction

The sympathetic nervous system serves as one of the first mechanisms of compensation in response to cardiac injury but is also undoubtedly involved in the pathogenesis of heart failure (HF). Sustained catecholamine hyperstimulation contributes to cardiac hypertrophy and myocardial dysfunction and is a central component of HF with reduced ejection fraction (HFrEF), for which beta-adrenergic receptor (beta-AR) blockade is a proven therapy [1]. In contrast, this blockade approach lacks efficacy in HF with preserved ejection fraction (HFpEF), where treatment with a beta-AR agonist, albuterol, has been recently proven to improve pulmonary function and increase exercise cardiac output reserve [2]. Thus, the beta-AR signaling plays a determinant role in cardiac remodeling. Cardiac remodeling targets not only cardiomyocytes, with alterations of survival, geometry, contractile or electric activity, but also interstitial cells such as fibroblasts, endothelial cells, vascular smooth muscle cells, and immune cells, leading to fibrosis, alteration of angiogenesis, and inflammation processes.

Cardiac macrophages play a determinant role in maintaining cardiac homeostasis, driving reparative processes after injury or contributing to disease progression [3]. These plastic cells adapt their physiology in response to cardiac and systemic stimuli. It is clearly emerging that beta-AR pathways interplay with macrophages to play a pivotal role on inflammation and immunomodulation shaping the phenotype of tissue remodeling [4,5,6]. We recently demonstrated that hearts subjected to chronic beta-AR agonist (isoproterenol (Iso)) stimulation exhibit an early adaptive transient increase in tissue macrophages exerting a CX3CL1/TNFalpha-dependent pro-hypertrophic impact and a protective role against transition to HF [5]. In addition, another study by our group identified activation of the Orai3-dependent calcium channel in response to TNFalpha secreted by macrophages as a novel pro-hypertrophic stimulus in Iso- and pressure overload models of early cardiac remodeling [6].

Macrophages are crucial in controlling and regulating the local tissue microenvironment, the matrix, oxygen content, acidification, and other molecular components (e.g., cytokines, growth factors, chemokines) associated with microenvironmental shifts. Macrophage metabolic changes can, therefore, be used as an essential indicator for the detection of changes in tissue homeostasis [7]. In addition, it is now recognized that macrophage metabolism, including lipid metabolism, not only provides energy but also greatly influences their phenotype and function, for example modulating signal transduction and gene regulation [8]. In line with this, lipid synthesis modulates inflammatory responses and phagocytosis and fatty acid oxidation impacts bioenergetics [8]. Dysregulation of lipid metabolism in macrophages is associated with various diseases [8]. Thus, defining and characterizing macrophages during disease development using RNA sequencing and lipidomic approaches arises as a prerequisite for optimizing a macrophage-targeted therapeutic potential.

This paper aims at deeply evaluating the kinetics of Iso-induced changes in macrophages transcriptomics to highlight the progressive reprogramming of macrophages but also to uncover potential specific properties of macrophages isolated from early adaptive versus late failing hearts, based on a complementary lipidomic approach and an extensive literature analysis.

## 2. Material and Methods

### 2.1. Ethics

Care of the animals and surgical procedures were performed according to the Directive 2010/63/EU of the European Parliament, which had been approved by the Ministry of Agriculture, France, (authorization for surgery C-75-665-R). The project was submitted to the French Ethic Committee CEEA (Comité d’Ethique en Expérimentation Animale) and obtained the authorization Ce5/2012/050 and APAFIS#1729-2015-083114195840v8. All experiments were performed in accordance with relevant named guidelines and regulations and in compliance with the ARRIVE guidelines.

Isoflurane was used to anesthetize mice during Alzet micropump implantation (2–3%). The adequacy of anesthesia was confirmed by the absence of a reflex response to foot squeeze. Mice were euthanized via cervical dislocation and hearts were isolated for analyses described below.

### 2.2. Animals

Experiments were conducted on adult male C57BL/6J mice (11–13 week-old) purchased from Janvier Labs (Le-Genest-St-Isle, France).

### 2.3. In-Vivo Chronic Isoproterenol Infusion

Mice were implanted with an osmotic micropump (Alzet, Charles River, L’Arbresles, France) containing either Isoproterenol (Iso: 30 mg/kg/day) or vehicle for either 14 or 28 days to develop either ECH or HF, respectively, as previously reported [5,6]. As previously published, there was a variability in the kinetics or in the maximum intensity of cardiac remodeling triggered by Iso. For this reason, each Iso protocol included all groups (Ct, ECH, and HF) of animals to allow comparison. Animals were part of the same batch, had same age and received Iso preparation either for 14 or 28 days, sharing a common period of housing in the animal facility. 

### 2.4. Measurement of Cardiac Parameters

Echocardiography was performed on lightly anesthetized animals under isoflurane (0.2–0.5%) with a probe emitting ultrasounds from 9- to 14-MHz frequency (Vivid7 PRO apparatus; GE Medical System Co, GE Healthcare, Velizy-Villacoublay, France), as previously reported [6]. At the end of protocols, mice were characterized according to the following criteria:

− cardiac hypertrophy (ECH and HF groups) based on the HW/TL parameter;
− normal (ECH group) or altered (HF group) cardiac function based on the FS parameter;
− presence (HF group) of dilation based on the LVd parameter.

All ECH animals were sacrificed at day 14 with echography performed at either day 12 for mice used for lipidomic or day 14 for mice used for RNAseq experiments. Of note, results suggest that day 12 could be a more suitable average time-point to optimally evidence thickening parameters (PW, IVS, and h/r) in WT C57Bl6/J mice (Supplementary Materials Figure S2).

### 2.5. Preparation of Immune Cells for Fluorescence Activated Cell Sorting

After perfusion with PBS, the mice heart was excised and digested in HBSS medium containing 2.5 mg/mL collagenase D (Roche, Meylan, France) for 30 min at 37 °C, with stirring. Erythrocytes were lysed by using red blood cell lysis buffer (MiltenyiBiotec, Paris, France). As shown in Supplementary Materials Figure S1, Cardiac immune cells were isolated by centrifugation, enriched by immunoselection using an anti-CD45 antibody coupled to magnetic beads (MiltenyiBiotec, Paris, France). Samples were blocked with Fc block (Ebioscience, Paris, France) prior to labeling with antibodies. Cytometry data were acquired on a BD FACSAria II cell sorter. After gating on CD11b^+^ cells, doublets were excluded and live (PI exclusion) CD14^+^/CD64^+^ macrophages were sorted directly into RLT lysis buffer (Qiagen, Les Ulis, France) or water and kept at −80 °C until RNAseq or lipidomic analysis, respectively, as shown in Supplementary Materials Figure S1 and as previously reported [5].

### 2.6. Antibodies for Sorting 

Antibodies used for cell sorting are shown in Table 1.

### 2.7. RNA Sequencing and Statistical Analysis

Total RNA from FACS sorted CD64^+^/CD14^+^ cells was isolated using the Nucleospin RNA XS kit (Macherey Nagel, Hoerdt, France), according to the manufacturer instructions. cDNA libraries were generated using total RNA with SMART-Seq v4 Ultra Low Input RNA Kit (TAKARA) and constructed according to manufacturer protocols as previously reported [9]. Paired end sequencing (2 × 750 bp) was performed by Nextseq 500 machine using High Output kit (150 cycles). Raw sequencing data was quality-controlled with the FastQC program. Trimmomatic was used to remove adapter sequences, trim low-quality reads, and discard reads shorter than 40 bp. Reads were aligned to the mouse reference genome (build mm10) with the TopHat2 tool. Mapping results were quality-checked using RNA-SeQC. Aligned reads were counted using the FeatureCounts and Express software, at the gene-level and transcript-level, respectively. Normalization and differential statistical analysis were performed with the GLM EdgeR package. RNA-Seq data has been made publicly available through the NCBI Gene Expression Omnibus (GEO), GEO accession number GSE157035, as previously reported [5]. Gene Analyses were performed using Ingenuity Pathway Analysis (IPA) (version 448560M) (Qiagen, les Ulis, France) and Metascape software (developped by Zhou et al. [10]).

### 2.8. Lipidomic Analysis

#### 2.8.1. Materials for Lipidomic Analysis

A high number of sorted Cd14^+^/Cd64^+^ cells was required for lipidomic experiments (around 40,000 cells/assay instead of 10,000 for RNAseq). Thus, cells obtained from 2–6 animals were pooled before lipid extraction.

All internal standards were purchased from Avanti Polar Lipids (Alabaster, AL, USA). LC/MS grade or UPLC grade solvents were used without further purification and obtained from Sigma-Aldrich (St Louis, MO, USA).

#### 2.8.2. Extraction

Phospho- and sphingolipids were extracted using a modified Bligh and Dyer method. Samples supplemented with a mixture of internal standard were extracted with 1.2 mL methanol/CHCl_3_ (2:1 *v*/*v*) in the presence of the antioxydant BHT and 310 µL HCl 0.005 N. Phase separation was triggered by addition of 400 µL CHCl_3_ and 400 µL water. Extracted lipids were dried and resuspended in LC/MS solvent.

#### 2.8.3. LC/MS Analysis

Lipids were quantified by LC-ESI/MS/MS using a Prominence UFLC (Shimadzu, Tokyo, Japan) and QTrap 4000 mass spectrometer (AB Sciex, Framingham, MA, USA) equipped with a turbo spray ion source (450 °C) combined with an LC20AD HPLC system, a SIL-20AC autosampler (Shimadzu, Kyoto, Japan) and the Analyst 1.5 data acquisition system (AB Sciex, Framingham, MA, USA). 

Quantification of phospholipids, sphingolipids and neutral lipids was performed in positive-ion mode. Sample (4 µL) was injected to a Kinetex HILIC 2.6 µm 2.1 × 150 mm column (Phenomenex, CA, USA). Mobile phases consisted of water and acetonitrile containing ammonium acetate and acetic acid. Lipid species were detected using scheduled multiple reaction monitoring (sMRM). N_2_ was used as nebulization and collision gas. Air was used as exhaust gas.

#### 2.8.4. Quantification

Lipids were quantified using thirty-seven calibration curves specific for the 16 individual lipid subclasses and up to 12 fatty acid moieties. More abundant lipid species which displayed non-linear response in non-diluted extracts were quantified from a 20-fold diluted sample.

#### 2.8.5. Isotope Correction of MRM Spectra

An in-house developed R script was used to correct for Isotopic contribution to MRM signals and adapted from Ejsing CS et al. [11].

### 2.9. Statistics Analysis

Echocardiographic, hypertrophic remodeling and lipidomic data analysis was performed with GraphPad Prism 8 (GraphPad software Inc., San Diego, CA, USA), using Kruskal–Wallis followed by Dunn’s post-hoc test. Quantitative data are reported as means ± SEM. For RNAseq data, normalization and differential statistical analysis were performed with the glm edgeR package. Additional comparison between ECH and HF groups was performed with GraphPad Prism 8, using Mann–Whitney test.

## 3. Results

**Figure 1:**
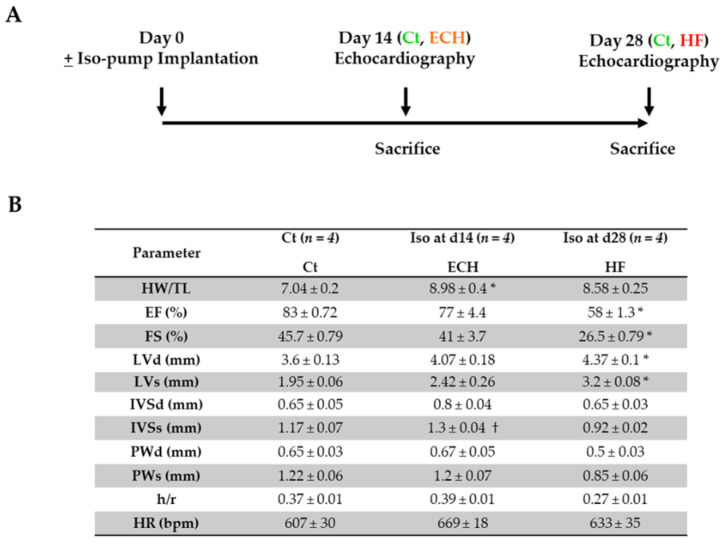
Schematic representation of iso-pump protocol with time-lapse of echocardiographic and morphologic measurements (**A**) and echocardiography and morphology parameters (**B**). HR, heart rate; IVSd, end-diastolic interventricular septum thickness; LVd, end-diastolic left ventricular diameter; PWd, end-diastolic posterior wall thickness; IVSs, end-systolic interventricular septum thickness; LVs, end-systolic left ventricular diameter; PWs, end-systolic posterior wall thickness; h/r, diastolic wall thickness to radius ratio; EF, ejection fraction; FS, fractional shortening; HW/TL, heart weight/tibia length. Kruskal–Wallis followed by Dunn post-hoc tests. * *p* < 0.05 ECH or HF vs. Ct; † *p* < 0.05 ECH vs. HF.

As recently reported [5,6], cardiac remodeling was induced in 11–13 week-old male C57BL/6J mice by chronic isoproterenol infusion (30 mg/kg/day) for 14 days or 28 days (Figure 1A).

Mice (*n* = 4/group) were characterized according to the following criteria: cardiac hypertrophy (ECH and HF groups) based on the HW/TL parameter, normal (ECH group) or altered (HF group) cardiac function based on the FS parameter and presence (HF group) of dilation based on the LVd parameter (Figure 1B).

**Figure 2:**
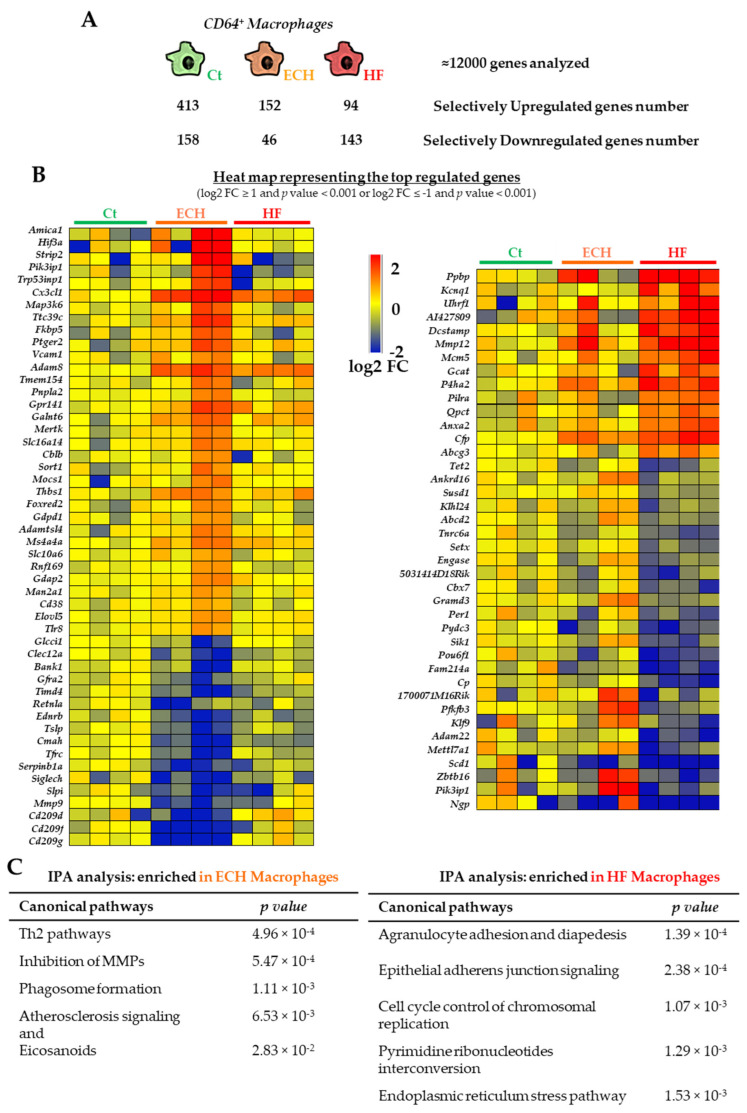
Transcriptomic characterization of cardiac Ct, ECH, and HF CD64^+^ macrophages. (**A**) Number of selectively upregulated and downregulated genes in Ct, ECH and HF macrophages. (**B**) Heat map showing the top genes selectively regulated in ECH (**left**) and HF (**right**) macrophages as compared to Ct and HF or Ct and ECH counterparts, respectively (with red and blue indicating increased and decreased expression, respectively). *n* = 4 mice/group. Normalization and differential statistical analysis were performed with the glm edgeR package. (**C**) RNAseq analysis. Ingenuity Pathway analysis showing specific canonical pathways and functions statistically enriched in ECH (**left**) and HF (**right**) macrophages. Part of the results concerning ECH macrophages specificities were published in Flamant et al.

Transcriptomic data analyzed in this study were obtained from the RNA sequencing of cardiac CD64^+^ macrophages (gating strategy described in Supplementary Materials Figure S1) isolated from Ct, ECH, and HF mice, as previously published [5]. This initial study by Flamant et al., focused on ECH macrophages and the role of the CX3CL1/CX3CR1 axis in the development of ECH. The present study aimed at performing an in-depth comparison between Ct, ECH, and HF global transcriptomic profiles to highlight ECH or HF macrophages signatures versus genes whose expression was progressively impacted by Iso treatment (RNAseq data publicly available through the NCBI Gene Expression Omnibus (GEO), GEO accession number GSE157035). Using a threshold of twofold change and false discovery rate (FDR) < 0.05, RNAseq identified 413, 152, or 94 genes selectively upregulated and 158, 46, or 143 genes selectively downregulated, in Ct, ECH, or HF macrophages, respectively (Figure 2A).

Figure 2B shows the heat map illustrating selective ECH and HF regulated top genes (as compared to Ct and HF or Ct and ECH counterparts, respectively) and Figure 2C the associated IPA analysis of canonical pathways. As expected, ECH hearts displayed a higher number of upregulated genes whereas HF hearts were characterized by a predominance of downregulated genes. Of note only part of ECH results was previously included in Flamant et al., with few genes being briefly commented [5]. Metascape analyses of enriched ontology clusters among ECH (A) and HF (B) selective genes are shown in Supplementary Materials Table S1. They highlight tissue remodeling, positive regulation of apoptotic processes and inflammatory response in ECH versus double-strand break repair and cell growth in HF macrophages.

Based on these initial results, we then performed an in-depth analysis of the literature concerning the major pathways identified in order to highlight the evolution of macrophages transcriptomics during beta-adrenergic- induced remodeling.

### 3.1. Genes Associated with Beta-Adrenergic Signaling

**Figure 3:**
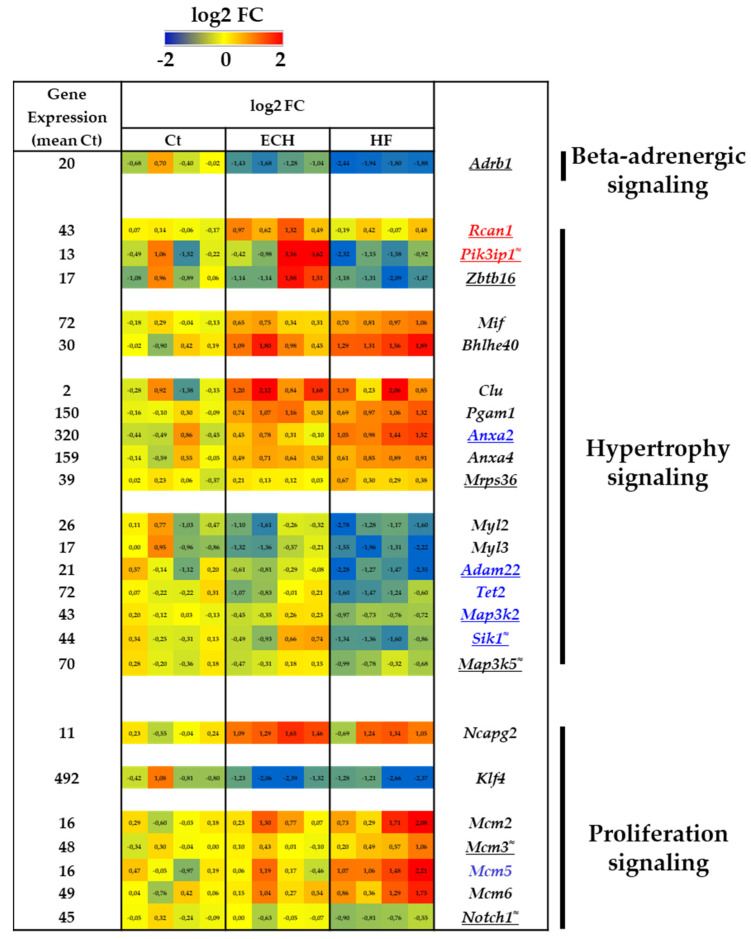
Transcriptomic characterization of Ct, ECH, and HF cardiac CD64^+^ macrophages. Beta--adrenergic--**,** hypertrophy--, and proliferation--signaling related genes. Analysis of mRNA levels in isolated cardiac CD64^+^ macrophages by RNAseq (*n* = 4 mice/group), normalization and differential statistical analysis were performed with the glm edgeR package, with genes selectively and significantly regulated in the ECH group (written in red) as compared to Ct and HF and significantly regulated in HF group (written in blue) as compared to Ct and ECH. Genes written in black are compared to Ct and genes underlined display statistical different expression between ECH and HF groups (*p* < 0.05 Mann–Whitney test or addition of the symbol ≈ means *p* = 0.05 HF vs. ECH). (**Left column**) Indication of the mean value of gene expression in Ct macrophages (expressed in counts per million mapped reads (cpm) estimates). (**Middle columns**) Heat map representation of log2 fold change. (**Right column**) Significantly regulated genes.

Isoproterenol (Iso) treatment resulted in macrophages gene expression changes downstream of beta-adrenergic receptor signaling with a decrease in *Adrb1* (coding for the beta_1_-AR), as previously reported in cardiac homogenates [12] (Figure 3), but no change in *Adrb2* (coding for the beta_2_-AR) (not shown).

### 3.2. ECH and HF Macrophages Are Characterized by Expression of Tissue Remodeling Genes Related to Cardiac Hypertrophy

Surprisingly, a number of genes previously found to be regulated in cardiac homogenates and related to Iso-induced cardiomyocyte hypertrophy [13] were identified in cardiac macrophages (Figure 3). Of note, the atypical expression of marker genes in ‘unrelated’ cell type has already been evidenced in other studies [14]. 

As compared to Ct and HF hearts, ECH macrophages, isolated from hypertrophic hearts with compensated function, were characterized by a selective transient induction of *Rcan1* (Calcipressin 1) and *Pik3ip1* (Phosphoinositide 3 kinase interacting protein 1). *Zbtb16* (Promyelocytic leukemia zinc finger protein, PLZF) was also elevated in ECH vs. HF hearts. Of note, these genes were reported as positive regulators of early (*Zbtb16*) or physiological (*Pik3ip1*) hypertrophic responses, or direct negative regulator of pathologic hypertrophy and HF (*Rcan1*) [15,16,17,18,19].

Similarly, Iso-infusion induced a progressive regulation of *Mif* (Macrophage migration inhibitory factor) and *Bhlhe40* (Class E basic helix-loop-helix protein 40), previously associated with a limitation of pathological hypertrophy [20,21]. 

Some pro-hypertrophic genes were increased in ECH macrophages and remained elevated in HF macrophages (e.g., *Clu* (Clusterin) and *Pgam1* (Phosphoglycerate mutase 1)) [13]. Other pro-hypertrophic genes displayed a progressive elevation between Ct, ECH, and HF macrophages such as *Anxa2* (Annexin 2) proposed as a biomarker of human HF [22], *Anxa4* (Annexin 4), and *Mrps36* (28S ribosomal protein S36, mitochondrial) [13].

HF macrophages, isolated from hypertrophic hearts with altered function, displayed a downregulation of several anti-hypertrophic factors such as *Myl2* and *Myl3* (myosin regulatory light chain 2 and 3), *Adam22* (disintegrin and metalloproteinase domain-containing protein 22) and *Tet2* (methylcytosine dioxygenase) [23,24,25,26]. In contrast, HF macrophages showed also a selective reduction of pro-hypertrophic signaling markers such as *Map3k2* (mitogen activated protein kinase kinase kinase 2, MEKK2) [27] and *Sik1* (salt inducible kinase 1) [28], as well as *Map3k5* (mitogen activated protein kinase kinase kinase 5, ASK1), a reported determinant factor in Angiotensin 2- and aldosterone/salt-induced cardiac hypertrophy [25,29].

Thus, different kinetic signatures of genes associated with hypertrophy are highlighted in macrophages, suggesting distinct, Iso-induced, early, transient, or late, transcriptomic programs.

### 3.3. Induction of Proliferation Programs in ECH and HF Macrophages

We previously demonstrated an increase in macrophages proliferation in Iso-induced ECH hearts (using Ki-67 immunostaining and BrdU incorporation experiments) [5] and our transcriptomic analysis revealed an associated early stimulation of the cell cycle modulator and proliferative marker *Ncapg2* (Condensin 2 complex subunit G2) [30]. In contrast, the expression of the inducer of macrophages proliferation, *Klf4* (Krüppel-like factor 4), previously identified in the transverse aortic constriction (TAC) model [31], was downregulated in response to Iso. This could suggest that Iso-induced macrophages proliferation operates independently of macrophages *Klf4* induction, a process that has been proposed to be driven by renal CSF2 (colony stimulating factor 2) in the TAC model [31].

HF macrophages display high levels of several members of the mini-chromosome maintenance complex, MCM related genes (*Mcm2, Mcm3, Mcm5, Mcm6*), initiators of genome replication, cell cycle progression and key triggers of cell proliferation (Figure 3) [32,33]. A parallel downregulated expression of Notch1 (encoding Neurogenic locus notch homolog protein 1) was detected. Of note, the NOTCH pathway is a negative regulator of MCM proteins expression [34].

Taken together, these results could suggest an increased proliferative activity of both ECH and HF cardiac macrophages, potentially occurring via distinct signaling mechanisms.

### 3.4. Time-Dependent Beta-Adrenergic-Induced Regulation of Inflammation, Fibrosis, Phagocytosis, Angiogenesis, and Antigen Presentation Genes in Macrophages

#### 3.4.1. ECH Macrophages Display Specific Anti-Inflammatory, Reparative, Pro-Phagocytic, and Pro-Angiogenic Transcriptomic Characteristics

**Figure 4:**
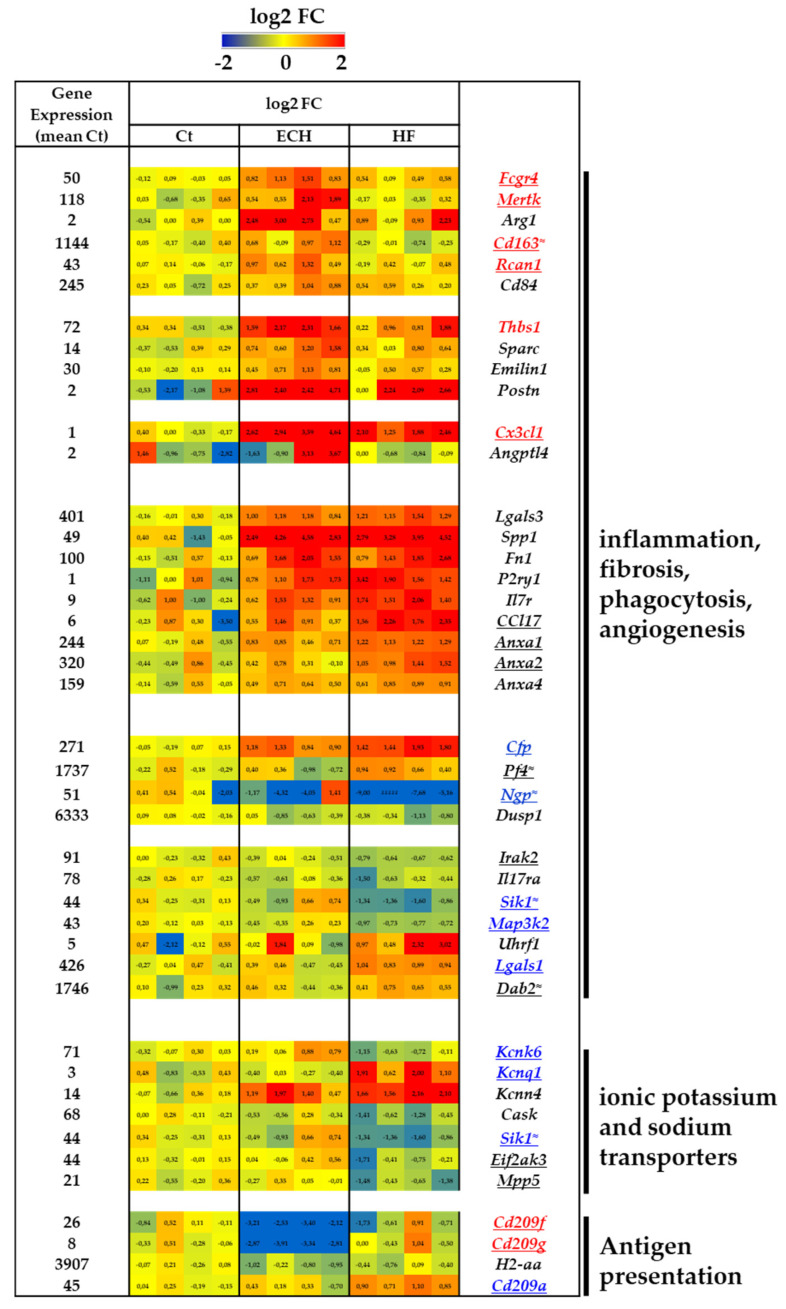
Transcriptomic characterization of inflammation, fibrosis, phagocytosis, angiogenesis, and Ab presentation related genes in Ct, ECH, and HF cardiac CD64^+^ macrophages. Analysis of mRNA levels in isolated cardiac CD64^+^ cells by RNAseq (*n* = 4 mice/group, normalization and differential statistical analysis were performed with the glm edgeR package, with genes selectively and significantly regulated in the ECH group (written in red) as compared to Ct and HF and significantly regulated in HF group (written in blue) as compared to Ct and ECH. Genes written in black are compared to Ct and genes underlined display statistical different expression between ECH and HF groups (*p* < 0.05 Mann–Whitney test or addition of the symbol ≈ means *p* = 0.05 HF vs. ECH). (**Left column**). Indication of the mean value of gene expression in Ct macrophages (expressed in counts per million mapped reads (cpm) estimates). (**Middle columns**). Heat map representation of log2 fold change. (**Right column**). Significantly regulated genes.

ECH macrophages were characterized by an upregulation of typical anti-inflammatory markers (Figure 4), among which *Fcgr4* (low affinity immunoglobulin gamma Fc region receptor IV, equivalent for human CD16-2), *Mertk* (tyrosine protein kinase Mer), *Arg1* (arginase 1), *Cd163* (scavenger receptor cysteine rich type 1 protein M130), *Rcan1* (previously listed as hypertrophic marker (Figure 3)) or *Cd84* (signaling lymphocytic activation molecule 5, Slamf5) [35,36].

Associated with this anti-inflammatory profile, ECH macrophages were enriched in fibrosis-related extracellular matricellular genes such as *Thbs1* (TSP1, thrombospondin), *Sparc* (Secreted protein acidic and rich in cystein), *Emilin1* (Elastin microfibril interface located protein 1), and *Postn* (Periostin) (Figure 4). These genes were previously reported to protect the failing heart from adverse remodeling and dysfunction (*Thbs1* and *Sparc*) or involved in collagen maturation and matrix production in reparative post-myocardial infarction (MI) (*Emilin1* and *Postn*) [37].

As discussed above, ECH macrophages displayed a selective increase in *Mertk* (Figure 4). MERTK is associated with anti-inflammatory and phagocytic macrophages functions and its determinant role has recently been described in resident macrophages to ensure elimination of cardiomyocyte extruded mitochondria-laden exophers and preserve metabolic stability and ventricular function [38].

Genes related to the regulation of angiogenesis, such as *Thbs1*, *Sparc*, *Cx3cl1* (Fractalkine), and *Angptl4* (Angiopoietin related protein 4) were also upregulated in ECH macrophages (Figure 4) [39,40].

#### 3.4.2. Early but Persistent Induction of Tissue-Resident-Related Protective Genes in Response to Iso

Other genes, described in the literature as related to tissue-resident macrophages and presenting properties similar with those identified above in ECH macrophages, were upregulated in ECH and further amplified in HF macrophages. This was the case of pro-fibrotic and/or anti-inflammatory *Lgals3* (galectin-3), *Spp1* (osteopontin), *Fn1* (fibronectin1), *P2ry1* (purinergic receptor), *Ccl17* (C-C motif chemokine 17), *Anxa1*, *Anxa2* (previously listed in hypertrophic markers), and *Anxa4* [37,41,42,43,44,45,46,47,48,49,50] (Figure 4). Of note, Lgals3 is currently developed as a new generation diagnostic marker for detecting the early stages of various heart diseases [41,42]. Recently related to Lgals3 [42], *Spp1* is involved in macrophages polarization and phagocytosis [37] and *Fn1* is an injury-associated matrix macromolecule [43,44]. *P2ry1* and *Il7r* are related to M2-polarization [45] and tissue resident macrophage development [46], respectively. Similarly the M2 chemokine *Ccl17* [47], coding for a ligand of CCR4, has been detected in early TAC-induced cardiac remodeling [48]. ANXA1 is an anti-inflammatory and pro-resolving mediator [49] and ANXA2 is associated with pro-angiogenic properties of macrophages [50].

#### 3.4.3. Moderate Evolution towards a Pro-Inflammatory Gene Program Characterizes HF Macrophages

As compared to Ct and ECH counterparts, HF macrophages were characterized by an induction of pro-inflammatory genes related to adverse remodeling such as *Cfp* (PROPERDIN) and *Pf4* (Platelet factor 4, CXCL4) (Figure 4). Consistent with the reported activation of the system complement in HF [51,52], PROPERDIN is a positive regulator of the alternative complement pathway [53]. CXCL4 is an anti-angiogenic chemokine [54] limiting phagocytic macrophage activity [55] and favoring adverse remodeling [54,55,56]. In addition, HF macrophages exhibited a marked downregulation of anti-inflammatory markers among which *Ngp* (Neutrophilic granule protein) [57] and *Dusp1* (Dual specificity protein phosphatase 1) [58] (Figure 4).

**Figure 5:**
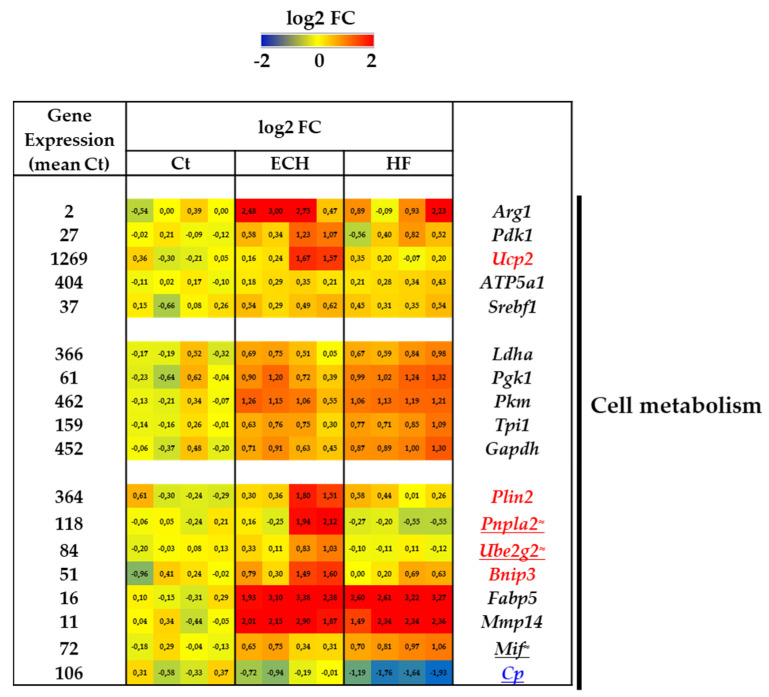
Transcriptomic characterization of Ct, ECH, and HF cardiac CD64^+^ macrophages. Cell metabolism related genes. Analysis of mRNA levels in isolated cardiac CD64^+^ cells by RNAseq (*n* = 4 mice/group, normalization and differential statistical analysis were performed with the glm edgeR package, with genes selectively and significantly regulated in the ECH group (written in red) as compared to Ct and HF and significantly regulated in HF group (written in blue) as compared to Ct and ECH. Genes written in black are compared to Ct and genes underlined display statistical different expression between ECH and HF groups (*p* < 0.05 Mann–Whitney test or addition of the symbol ≈ means *p* = 0.05 HF vs. ECH). (**Left column**). Indication of the mean value of gene expression in Ct macrophages (expressed in counts per million mapped reads (cpm) estimates). (**Middle columns**). Heat map representation of log2 fold change. (**Right column**). Significantly regulated genes.

However, HF macrophages also displayed a decrease in several M1-like markers (Figure 5) among which *Irak2* (Interleukin 1 receptor associated kinase like 2), *Il17ra*, *Sik1* (cited above in hypertrophic markers), and *Map3k2* (MEKK2) [59] (Figure 4). Of note, SIK1 inhibition not only limits inflammation [60,61] but also reduces adverse cardiac remodeling [28]. A reduction of proinflammatory *Map3k2* potentially restrains the cardiac hypertrophic response [27]. In addition, HF macrophages were characterized by an induction of anti-inflammatory markers such as *Uhrf1* (E3 ubiquitin protein ligase), *Lgals1* (galectin 1), and *Dab2* (disabled homolog 2). *Uhrf1* is an epigenetic regulator that represses TNFalpha expression [62]. *Lgals1* is an emerging mediator that tempers cardiovascular acute and chronic inflammation [63]. *Dab2* is a regulator of phenotypic switching in macrophages, increased in M2 macrophages [64].

These results could suggest the maintenance of a relatively anti-inflammatory environment in HF macrophages, despite a tendency towards an elevation of inflammatory response when compared to ECH counterparts. 

#### 3.4.4. Typical Regulation of K^+^ and Na^+^ Transport Genes in HF Macrophages

A number of genes related to ion transport were modulated in HF macrophages (e.g., Kcnk6 (coding for the K+ channel efflux TWIK2), Kcnq1, Kcnn4, Cask (calcium/calmoduline dependent serine protein kinase), Eif2ak3 (PERK), Sik1 and Mpp5 (MAGUK)) (Figure 4). Inhibition of Kcnk6 in HF macrophages was potentially protective since Kcnk6 is major actor in NLRP3-inflammasome activation [65] promoting adverse cardiac remodeling in the TAC model [66]. In contrast, induction of Kcnn4 was potentially associated with facilitated inflammation and promotion of fibrosis as described in AngII treated rats [67] or in response to beta-adrenergic stimulation [68]. Of note, Kcnn4, upregulated in M1 macrophages, facilitates cardiac arrhythmias, regulating cardiomyocyte electrical conduction via gap junctions in the MI border zone [69]. 

HF macrophages were also characterized by a downregulation of *Cask*, *Sik1*, *Eif2ak3*, and *Mpp5*, four negative regulators of cardiac sodium channel Nav1.5 in cardiomyocytes [70,71,72]. The resultant potential phagocyte Nav1.5 activation could exert a pro-inflammatory impact in HF hearts, since inhibition of Nav1.5 has been described as an anti-inflammatory strategy [73,74] improving post-infarction remodeling [73].

#### 3.4.5. Dendritic Cell Markers and Antigen Presentation in HF Macrophages

The downregulation of *Cd209f* and *Cd209g* dendritic cell markers and *H2-aa* (histocompatibility Antigen) expression as compared to control and HF macrophages argued for a limited antigen presentation potential of ECH macrophages. In contrast, HF macrophages were potentially enriched in Ag-presenting dendritic cells since they displayed an upregulated *Cd209a* expression (Figure 4) [75]. 

Taken together these results suggest that both ECH and HF macrophages exhibit overlapping activation of anti- and pro-inflammatory transcriptomic programs with a dominant anti-inflammatory tendency in ECH versus HF macrophages. Of note, in line with a limited role of recruited pro-inflammatory monocytes [5], the activation of pro-inflammatory genes is mitigated in Iso-infused as compared to TAC-induced HF macrophages. ECH macrophages were characterized by an induction of pro-fibrotic, pro-phagocytic and pro-angiogenic markers and HF macrophages displayed special regulation of ion channel related genes. 

### 3.5. Regulation of Macrophages Metabolism Associated with Beta-Adrenergic-Induced Cardiac Remodeling

Immunometabolism recently emerged as a central regulator of macrophage functions [76]. It is reported that anti-inflammatory (M2) macrophages mediate the resolution of inflammation and tissue repair, shifting their metabolism to fatty acid oxidation and oxidative phosphorylation. The M2 polarization exerts a switch on arginine metabolism [77] and fatty acid lipid synthesis [78]. Of note, mitochondrial metabolism plays an important role in regulating the inflammatory phenotype of macrophages. The M2 phenotype mainly depends on oxidative phosphorylation and characterized by an increased oxygen consumption [59]. The M1-like macrophages phenotype is associated with high glycolysis and minimal mitochondrial oxidative phosphorylation.

In keeping with such adaptation, predominant anti-inflammatory ECH macrophages exhibited a typical increase in *Arg1* (Figure 5).

In addition, ECH macrophages were characterized by an increased expression of *Pdk1* (pyruvate deshydrogenase) coding for a rate limiting enzyme of glucose oxidation [79]. ECH macrophages also exhibited an induction of *Ucp2* (mitochondrial uncoupling protein 2), that plays a cardioprotective role in cardiac hypertrophy promoting mitochondrial fission, ATP synthesis and a decreased ROS production [80] (Figure 5).

The mitochondrial complexe gene, *ATP5a1* (ATP synthase subunit alpha) was increased in ECH macrophages and maintained elevated in HF counterparts, in favor of a sustained mitochondrial oxidative function. Iso also induced a progressive induction of *Srebf1* (sterol regulatory element binding protein 1), involved in lipogenesis [59] (Figure 5).

In contrast, several genes associated with glycolysis were progressively upregulated in response to Iso or increased in ECH macrophages and maintained elevated in HF macrophages (Figure 5), among which *Ldha* (lactate deshydrogenase), *Pgk1* (phosphoglycerate kinase 1), *Pkm* (pyruvate kinase muscle isoform), *Tpi1* (Triose phosphate isomerase 1), and *Gapdh* (glyceraldehyde-3 phosphate deshydrogenase).

Lipid droplets (LDs) are ubiquitous organelles specialized in neutral lipid storage (i.e., tri- and di-acylglycerols and sterol esters), and their degradation (lipophagy) plays a pivotal role in the mobilization of fatty acids and cholesterol for energy production and cholesterol efflux [81]. ECH macrophages were enriched in *Plin2* (perilipin 2), *Pnpla2* (patatin-like phospholipase domain containing 2), and *Ube2g2* mRNA (ubiquitin conjugating enzyme E2 G2) (Figure 5). PLIN2 is one of the most abundant structural protein on the surface of LDs and PNPLA2 a lipase associated to LDs [82]. *Ube2g2* is a putative lipophagy promoting signal [82] and a regulator of cholesterol efflux [81]. ECH macrophages were also characterized by an induction of *Bnip3* (BCL2/adenovirus E1 B interacting protein 3) involved in mitophagy and potentially exerting cardioprotective survival effects [83].

*Fabp5* coding for the Fatty acid binding protein 5, involved in lipid transport and a potential protective mechanism against pathological remodeling [84], was markedly elevated in ECH and HF macrophages, as compared to Ct counterparts (Figure 5). A similar regulation was detected for genes such as *Mmp14* (matrix metalloproteinase 14), a candidate lipophagy regulator [81] and *Mif* (macrophage migration inhibitory factor), a cardioprotective activator of autophagy reported to mitigate pathological hypertrophic responses [85] (Figure 5). Cp (ceruloplasmin), a potent catalyst of LDL oxidation [86] and proposed biomarker of HF [87] was progressively downregulated in Iso-treated macrophages (Figure 5).

#### Enrichment in Lipid Signaling Characterizes Beta-Adrenergic-Induced ECH Macrophages

**Figure 6:**
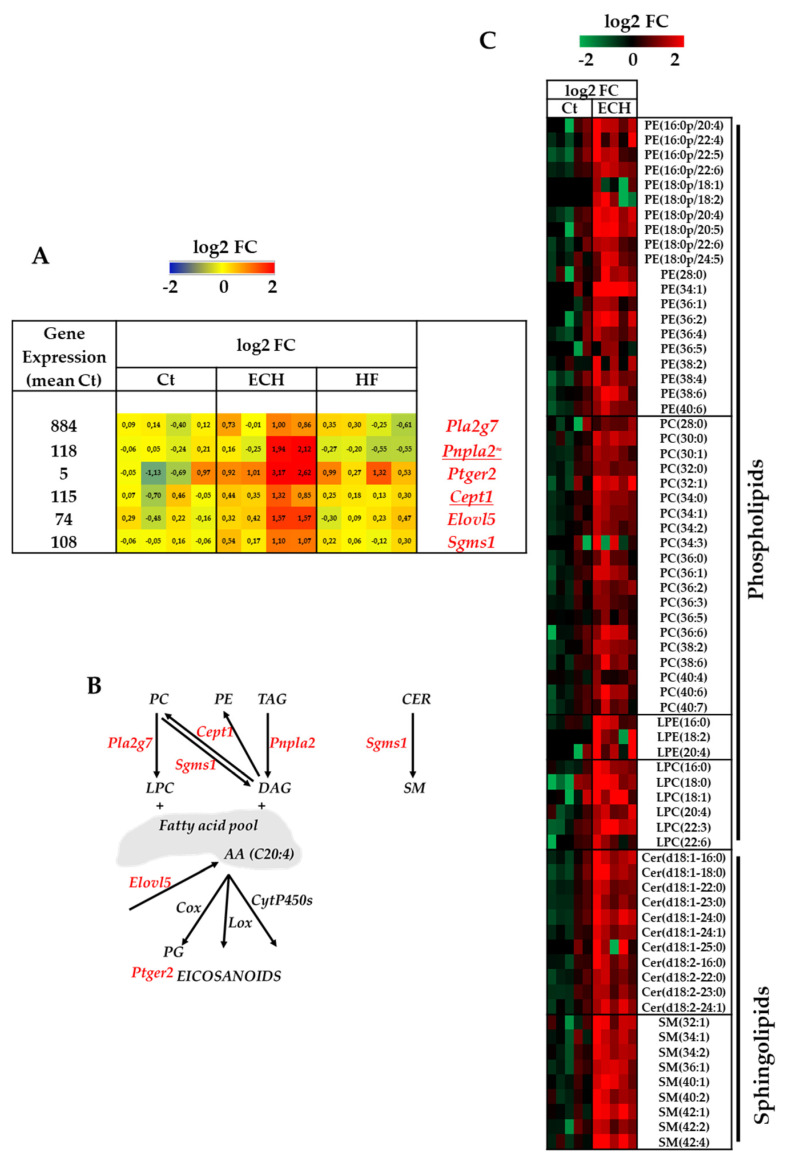
ECH macrophages are characterized by a net enrichment in lipid regulatory pathways related with eicosanoids, phospholipids, and sphingolipids metabolism. (**A**) RNAseq analysis of lipid regulatory pathway gene expression. *n* = 4 mice/group. Normalization and differential statistical analysis were performed with the glm edgeR package, with genes selectively and significantly regulated in the ECH group (written in red) as compared to Ct and HF. Genes underlined display statistical different expression between ECH and HF groups (*p* < 0.05 Mann–Whitney test or addition of the symbol ≈ means *p* = 0.05 HF vs. ECH). (**Left column**). Indication of the mean value of gene expression in Ct macrophages (expressed in counts per million mapped reads (cpm) estimates). (**Middle columns**). Heat map representation of log2 fold change. (**Right column**). Significantly regulated genes. (**B**) Graphical abstract of associated lipid pathways. (**C**) Lipidomic characterization of cardiac CD64^+^ macrophages from Iso-induced ECH versus control hearts (*n* = 5/group) sorted as previously described (Flamant et al., 2021, [5]). Kruskal–Wallis followed by Dunn’s post-hoc test. Cer, ceramide; LPC, lysophosphatidylcholine; LPE, lysophosphatidylethanolamine; PC, phosphatidylcholine; PE, phosphatidylethanolamine; PG, phosphatidylglycerol; PI, phosphatidylinositol; SM, sphingomyelin; TAG, triacylglycerides; DAG, diacylglycerides; Cox, cyclooxygenases; Lox, lipooxygenases; CytP450s, cytochromes P450; PG, prostaglandin; AA, arachidonic acid. Data were normalized to cell counts.

IPA highlighted an enrichment for pathways involved in atherosclerosis and eicosanoid signaling in ECH macrophages (Figure 2C). In fact, a hallmark of ECH macrophages was an enrichment in the expression of several lipid metabolism-related genes among which *Pla2g7* (Platelet activating factor acetylhydrolase), *Pnpla2* and *Ptger2* (Prostaglandin E2 receptor EP2 subtype), involved in arachidonic acid (AA) metabolism and prostaglandin signaling (Figure 6A,B).

A net increase in several lipid species was detected in ECH macrophages using a lipidomic analysis approach (Figure 6C). In agreement with the increased choline ethanolamine phosphotransferase 1 (*Cept1*) mRNA expression, ECH macrophages displayed higher amounts of several phosphatidylethanolamine (PE) and phosphatidylcholine (PC) molecular species, as compared to Ct macrophages (Figure 6A–C). The elevation in PE-plasmalogens was in favor of monocyte to macrophages terminal phagocytic differentiation [88]. It was associated with an induction of *Elovl5* (elongation of very long chain fatty acids protein 5) mRNA expression, also previously suggested as a potential biomarker of macrophages differentiation [88] and involved in long-chain polyunsaturated fatty acids biosynthesis and AA production (Figure 6A,B). Higher levels of several lysophosphatidylethanolamine (LPE) and lysophosphatidylcholine (LPC) lipid species in ECH cells also suggested an increase in PE and PC hydrolysis and a potential increased release of polyunsaturated fatty acids (PUFAs) contributing to eicosanoid signaling (Figure 6B,C). Of note, plasmalogens in the plasma membrane represent cellular stores for precursor molecules of eicosanoid biosynthesis, mainly AA [89]. 

In addition, ECH macrophages were characterized by an increased *Sgms1* mRNA expression (coding for the SMS1 protein), an enzyme that converts ceramides into sphingomyelins (Figure 6A,B). In line with this, ECH macrophages display elevated levels in several types of SM lipid species (Figure 6C). Several studies have reported a correlation between apoptotic resistance and increased SMS activity, with a change in the cellular balance between pro-apoptotic ceramide and anti-apoptotic sphingomyelins [90,91,92] and sphingosine-1-phosphate [93]. In addition, SMS1 plays a critical role in cell growth, e.g., of mouse lymphoid cells [94] and proliferation [95]. Thus, an increased SMS activity in ECH macrophages could favor cell survival and proliferation and partly drive the macrophages accumulation observed in ECH hearts [5]. 

Taken together, our RNAseq analysis suggests that ECH macrophages display an induction of metabolic genes involved in oxidative mitochondrial phosphorylation, glucose and fatty acid oxidation, lipophagy and eicosanoid signaling, and arginine metabolism. Our lipidomic approach further argues for an important modulation of lipid metabolism in ECH macrophages. Despite a tendency to retain fatty acid oxidation and mitochondrial oxidative gene expression, HF macrophages display an upregulation of several glycolysis related genes. 

### 3.6. Beta-Adrenergic Regulation of Genes Involved in Signaling Pathway Networks

#### 3.6.1. Induction of Anti-Apoptotic and Pro-Survival Genes in ECH and HF Macrophages

**Figure 7:**
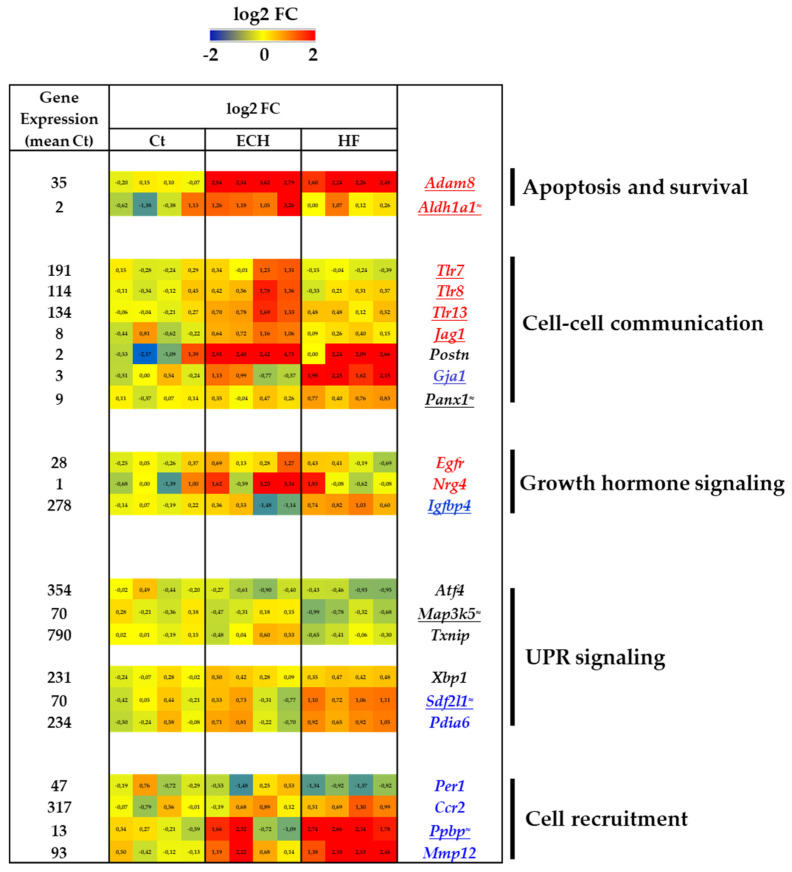
Transcriptomic characterization of Ct, ECH, and HF cardiac CD64^+^ macrophages. Apoptosis and survival, cell–cell communication, growth hormone signaling, UPR signaling, and cell recruitment related genes. Analysis of mRNA levels in isolated cardiac CD64^+^ cells by RNAseq (*n* = 4 mice/group, normalization and differential statistical analysis were performed with the glm edgeR package, with genes selectively and significantly regulated in the ECH group (written in red) as compared to Ct and HF and significantly regulated in HF group (written in blue) as compared to Ct and ECH. Genes written in black are compared to Ct and genes underlined display statistical different expression between ECH and HF groups (*p* < 0.05 Mann–Whitney test or addition of the symbol ≈ means *p* = 0.05 HF vs. ECH). (**Left column**). Indication of the mean value of gene expression in Ct macrophages (expressed in counts per million mapped reads (cpm) estimates). (**Middle columns**). Heat map representation of log2 fold change. (**Right column**). Significantly regulated genes.

ECH and HF macrophages displayed an increase in *Adam8* expression, an important biomarker in cardiovascular diseases [96], with protective proliferative, pro-survival, and anti-apoptotic properties (Figure 7) [97].

ECH macrophages were characterized by elevated *Aldh1a1* coding for the major enzyme catalyzing retinoic acid synthesis, a potent transcription activating hormone driving protective signaling pathways [98].

#### 3.6.2. Differential Regulation of Cell–Cell Communication Genes in ECH and HF Macrophages

ECH macrophages displayed an induction of several NOTCH-signaling related factors mRNA (e.g., *Tlr7*, *Tlr8*, *Tlr13*, and the Notch ligand *Jag1* (JAGGED1)) potentially playing a role in cell–cell communication and/or inflammatory polarization [99]. *Postn* gene upregulation, coding for a non-canonical Notch ligand previously related to human diastolic dysfunction [100], characterized ECH and HF macrophages (Figure 7). Of note, an alternative protective impact of NOTCH-signaling has been described in pressure overloaded heart due to limitation of fibrosis or in infarcted myocardium favoring angiogenesis [100].

Cardiac macrophages facilitate electrical conduction through CX43-dependent interaction with cardiomyocytes [101]. HF macrophages presented an upregulation of *Gja1* (coding for connexin 43). This gap junction protein has been associated with heart rate in HF patients [102], mitochondrial biogenesis and cardioprotection against IR injury [103], or inflammation [104]. *Panx1* (PANNEXIN 1), another cell communication-involved molecule was also upregulated (Figure 7).

#### 3.6.3. Induction of Growth Factor Signaling Genes in ECH Macrophages

Alternative wound repair macrophages are characterized by an enhanced production of several growth factors [105] and EGFR signaling is described as a cardiac specific macrophages signature [40]. In line with this, ECH macrophages display a transient upregulation of *Egfr* and *Nrg4* (pro-neuregulin 4) (Figure 7).

In contrast, HF macrophages were characterized by upregulated *Igfbp4* (insulin-like growth factor binding protein 4), a negative regulator of IGF1 signaling, proposed as a potential therapeutic strategy target for HF due to its cardiogenic properties [106]. Of note, the IGF1-PI3Kinase pathway plays an essential role in exercise-induced protective physiological hypertrophy [107].

#### 3.6.4. UPR Signaling in HF Macrophages

The unfolded protein response (UPR) signaling potentially limits apoptosis and inflammation in macrophages [108,109]. HF macrophages displayed an inhibition of the expression of several UPR genes such as *Atf4* (cyclic AMP dependent transcription factor), *Eif2ak3* (cited in ion transporters section), *Map3k5* (ASK1), *Txnip* (thioredoxin interacting protein) (Figure 7). However, they also exhibited an increase in *Xbp1* (Treb5), *Sdf2l1* (stromal cell derived factor 2 like protein 1), and *Pdia6* (protein disulfide isomerase A6), associated with protein folding and misfolded protein degradation [110,111]. Taken together these results could suggest a complex regulation of UPR signaling in HF macrophages.

### 3.7. Regulation of Genes Involved in Monocyte or Neutrophils Recruitment in HF Macrophages

HF macrophages displayed a downregulated expression of *Per1* (PERIODIN 1) and an increased expression of *Ccr2*. Of note, PERIODIN 1 is a circadian regulator protein that has been associated with limited recruitment of CCR2^+^ macrophages [112].

HF macrophages exhibited a net increase in *Ppbp* (CXCL7), a neutrophil chemoattractant [113] and reported biomarker of the severity of atherosclerosis (Figure 7) [114]. Of note, a combined elevation of *Cxcr2*, coding for the CXCL7 receptor was observed in blood samples of HF mice (unpublished data) arguing for a possible chemoattraction process. However, HF macrophages were also characterized by a marked induction of *Mmp12*, reported to limit neutrophils influx [115,116].

## 4. Discussion

The objective of this study was to perform a descriptive and integrative analysis of macrophages gene variations during cardiac remodeling. We did not aim to establish a causal relationship between the expression of these genes and the course of the disease. Nevertheless, among the many avenues that are suggested, our analysis may constitute the basis for more in-depth studies to identify important macrophage-related pathways interfering in cardiac remodeling or characterize biomarkers associated with early versus late disease progression.

**Figure 8:**
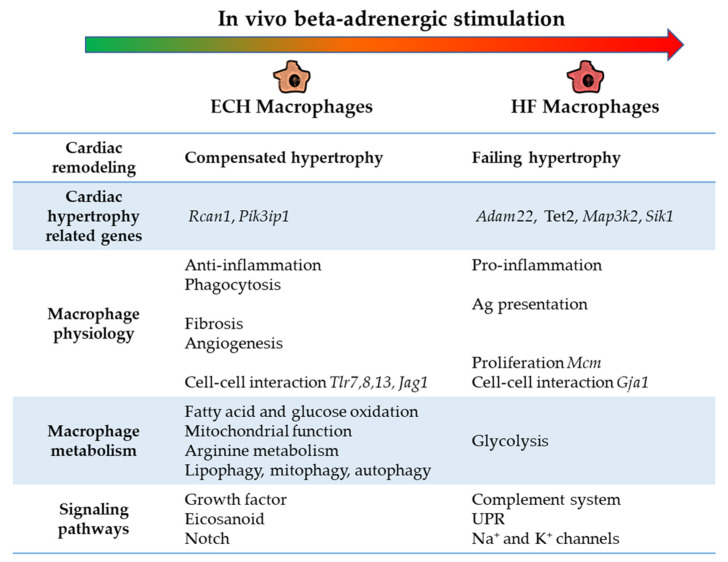
Specificities of ECH and HF cardiac CD64^+^ macrophages transcriptomic profiles.

We characterized the evolution of the molecular signature of cardiac macrophages in mice subjected to chronic beta-AR stimulation (Figure 8).

Our results point out both the potential novel properties of cardiac macrophages as well as new biomarkers of compensated versus failing remodeling (see graphical abstract).

First, we identified panels of hypertrophy-related genes regulated in ECH macrophages (*Rcan1*, *Pik3ip1*) or HF macrophages (*Adam22*, *Tet2*, *Map3k2*, *Sik1*) and thus potentially associated with compensated or failing hypertrophy remodeling, respectively [5]. A third series specify hypertrophy-related genes gradually regulated in response to Iso (such as *Mif*, *Bhlhe40*, *Clu*, *Pgam1*, *Anxa2*, *Anxa4*, *Mrps36, Myl2, Myl3*). In addition to emphasizing potential biomarkers of ECH versus HF cardiac remodeling, our results highlight within macrophages, an atypical induction of marker genes traditionally attributed to cardiomyocyte-specific hypertrophy. Atypical expression of marker genes in unrelated cell types has already been evidenced. In atherosclerosis, expression of the stem-cell marker pluripotency factor OCT4 in somatic vascular smooth muscle cells was found to exert a critical atheroprotective functional role [117]. During the progression of cardiac hypertrophy, expression of endothelial marker genes was described in cardiomyocytes [14]. *Myl2* expression was also detected in immune B cells. The notion of selective cell markers as well as tissue-specificity has evolved a lot in recent years and the potential for resident cardiac macrophages to express cardiac specific genes warrants to be further assessed.

Cardiac resident macrophages are established in neonates from primitive yolk sac and fetal monocyte lineages and persist into adulthood through self-renewal. Steady state resident macrophages proliferate in situ under physiological conditions [31]. They also exert cardioprotective compensatory functions in response to stress. Our results indicate that HF macrophages are characterized by induction of cell proliferation-related transcriptome programs, involving *Mcm* related genes. Of note, induction of *Klf4*, a key player of macrophages proliferation in TAC remodeling, is not detected, suggesting divergent regulations in the Iso model.

With aging and/or following heart injuries, the myocardium is progressively colonized by a second pool of macrophages replenished from circulating monocytes derived from hematopoietic precursors in the bone marrow or spleen [3,118]. Infiltrating monocytes and pro-inflammatory CCR2^+^ macrophages mainly contribute to exacerbated hypertrophy responses and fibrosis leading to HF [119]. Our previous flow cytometry results identified the predominant upregulated ECH macrophages subpopulation as Ly6c^low^/CCR2^-^/MHCII^low^ and suggested that CCR2^+^ macrophages play a negligible role in ECH-related hypertrophic remodeling [5]. ECH macrophages exhibit a dominant anti-inflammatory profile and are enriched in genes associated with phagocytosis. In line with this, CCR2^-^ macrophages display a 2.5-fold increase in apoptotic cell engulfment efficiency, as compared to their pro-inflammatory CCR2^+^ counterparts [118]. Our studies documented the dominant protective role of resident ECH macrophages in Iso-induced remodeling [5,6]: we demonstrated that clodronate depletion of ECH macrophages favors transition to HF in response to Iso [5]. In line with this, the depletion of CCR2^-^ cardiac resident macrophages before cardiac stress by Iso leads to increased mortality and fibrosis [38]. A progressive but modest elevation of *Ccr2* expression is detected in response to Iso together with the transition towards HF. This observation associated with our previous results argue for a limited participation of recruited CCR2^+^ monocytes in Iso-induced cardiac remodeling as compared to pressure-overload or ischemia-related models [3,5].

We identify a typical novel feature in HF macrophages, namely the potential regulation of voltage-gated Na^+^ and K^+^ transport-related genes. This underscores the need for future characterization of the electrophysiologic properties of resident cardiac macrophages during evolution towards HF, and consequences on their functions. In addition, HF macrophages display a more inflammatory profile and transcriptome modulation in favor of a lower phagocytic activity but higher Ag presentation capacity as compared to their ECH counterparts.

Transcriptomic and lipidomic results show that ECH macrophages are characterized by a typical lipid remodeling. Specifically, ECH macrophages display induction of *Pla2g7, Pnpla2*, and *Elovl5*, coding for enzymes potentially leading to AA production and *Ptger2*, an eicosanoid receptor encoding gene. PLA_2_ are involved in generation of lipid signaling molecules by hydrolysis of the sn-2 ester bound of glycerophospholipids to yield free long chain fatty acids (FA) and 2-lysophospholipids [120]. PLA_2_ are the upstream regulators of the eicosanoid pathway potentially liberating free AA from the sn-2 position of membrane phospholipids. AA can either function as an important signaling molecule or it can be oxidatively metabolized to various bioactive eicosanoids (including prostaglandins, thromboxanes, and leukotrienes) through cyclooxygenases (COX), lipooxygenases (LOX), and cytochrome P450s [120]. In the heart, eicosanoids exert important effects on receptor-, ion channel-, and transcription-mediated processes that facilitate cardiac hypertrophy, mediate ischemic preconditioning, activate inflammation or precipitate arrhythmogenesis in response to pathologic stimuli [121]. In cardiomyocytes, activation of a cytosolic PLA_2_/COX pathway via beta_2_-AR/Gi mediated stimulation has been suggested to participate in protective adaptive changes induced in the myocardium either by chronic intermittent hypoxia in rats, or in human HF [122,123,124,125]. In macrophages, AA and eicosanoids regulate immune cell activation, phagocytosis, host defense, inflammation, and the repair of membranes [126,127]. AA metabolites have been recently involved in the release of TNFalpha by monocytes [128] and peritoneal macrophages [129]. Interestingly, one of our recent studies reported that LOX-derived AA metabolites may drive a cardiac protective pro-hypertrophic effect in Iso-infused or TAC rats upon activation of Orai3-dependent calcium channels [6]. In the present study, IPA analysis clearly highlights induction of the eicosanoid signaling as a potential critical feature of protective ECH macrophages.

Our results indicate elevation of LPC species in ECH macrophages associated with induction of *Pla2g7*. Lp-PLA_2_ (encoded by *Pla2g7*) is secreted by macrophages with a favored substrate of oxidized phosphatidylcholine (PC), generating lyso-PC (LPC) and oxidized non-esterified fatty acids [130]. Lp-PLA_2_ can also hydrolyze platelet activating factor (PAF). It has been identified as a biomarker of cardiovascular disease, but clinical trials failed to identify evidence of a causal relationship between Lp-PLA_2_ activity and risk of CVD [130].

ECH macrophages display elevated *Pnpla2* expression. ATGL (encoded by *Pnpla2*) is a critical rate-limiting enzyme of lipolysis of triacylglyceride into DAG and free fatty acids (FFAs). ATGL regulates cardiac mitochondrial function via PPARs in non-immune cells and its global deletion results in cardiac insufficiency and lethal cardiomyopathy [131]. In line with this, patients with mutations in *Pnpla2* gene develop severe cardiomyovasculopathy [132]. In contrast, ATGL supports pro-inflammatory and chemotaxis responses in immune cells, contributing to the production of IL6 and AA (C20:4), the precursor of eicosanoids [133]. 

Interestingly, pharmacological or genetic inhibition of ATGL in adipocytes ameliorates Iso-induced cardiac inflammation, hypertrophy and fibrosis likely by reducing adipose tissue inflammation and reducing galectin-3 secretion from adipose tissue [134]. Of note our results indicate that *Pnpla2* induction in cardiac Iso ECH macrophages is associated with upregulation of *Lgals3* (coding for Galectin-3). Adipocyte ATGL deletion also attenuates the development of exercise-induced physiological cardiac hypertrophy [135], as well as TAC-induced cardiac hypertrophy and associated left ventricular phosphatidylethanolamine (PE) elevation [136]. In line with this, *Pnpla2* induction in cardiac Iso ECH macrophages was associated with increase in several PE species (among which PE(16:0–20:4) and PE(18:0–20:4), induced in the TAC model) [136]. Whether these regulations potentially resulted from direct Iso-induced intracardiac metabolic modifications or also derived from inter-organ communication (as previously illustrated in the TAC model [137] or documented by Smeir et al.) [138] remains an open question.

Our results strengthen previous findings concerning metabolic changes with cardiac remodeling. We show that beta-AR-induced cardiac remodeling triggers an important macrophages metabolic gene reprogramming. ECH cells exhibit upregulation of genes associated with Arginine metabolism, fatty acid and glucose oxidation and mitochondrial oxidative function, lipophagy, mitophagy, and autophagy. This is in keeping with a recent proteomic and metabolomic study showing that short term treatment of human U937 macrophages with Iso directly alters glucose metabolism by shifting it away from glycolysis [139]. In comparison, HF cells are enriched in genes involved in glycolysis. These metabolic orientations are in agreement with the current knowledge that metabolism drives macrophages function with oxidative phosphorylation and glycolysis promoting anti- and pro-inflammatory profiles, respectively. 

Our previous flow-cytometry experiments show that CD64^+^/CCR2^-^/Ly6C^low^/MHCII^low^ macrophages are increased in ECH but not in HF hearts, as compared to Ct. A limitation of our study is that our analysis indicates a time-dependent evolution of gene expression levels in iso-infused animals, highlighting early and late profiles. This suggests potential variation of macrophage functions with time but could also potentially reflect evolution in macrophage populations only. These observations are obtained from ECH or HF characterized animals but do not demonstrate any causal relations between transcriptomic profiles and heart phenotypes. However, our results provide a valuable resource for extending knowledge of macrophages biology and expanding the growing collection of molecular signatures of immune-related cells in different contexts and tissues. Given the key role of cardiac macrophages in cardiac remodeling and failure, this knowledge will likely benefit the future design of treatments for HF.

## Figures and Tables

**Table 1:** Antibodies used for cell sorting.

Experiment	Target	Clone	Isotype	Reference	Dilution	Fluorochrome	Source
sorting	CD64	REA-286	Human IgG1	130-103-808	1:40	PE	MiltenyiBiotec Paris, France
sorting	CD11b	M 1/70	Rat IgG2b, κ	48-0112-82	1:200	eFluor 450	Ebiosciences Paris, France
sorting	CD14	SA2-8	Rat IgG2a, κ	11-0141-82	1:200	FITC	Ebiosciences Paris, France

## Data Availability

RNA-Seq data has been made publicly available through the NCBI Gene Expression Omnibus (GEO), GEO accession number GSE157035, as previously reported [5].

## Supplementary Materials

The following supporting information can be downloaded at: https://www.mdpi.com/article/10.3390/biomedicines10020221/s1, Figure S1: Time-lapse of macrophage sorting and gating strategy for RNA sequencing and lipidomic analysis, Table S1: Transcriptomic characterization of ECH and HF cardiac CD64+ macrophages, Figure S2: Mice used in lipidomic analysis. Schematic representation of Iso-pump protocol with time-lapse of echocardiographic and morphologic measurements (A) and echocardiography and morphology parameters (B).
