# Supplementary material for: Transcriptomic and Lipidomic Mapping of Macrophages in the Hub of Chronic Beta-Adrenergic-Stimulation Unravels Hypertrophy-, Proliferation-, and Lipid Metabolism-Related Genes as Novel Potential Markers of Early Hypertrophy or Heart Failure
Source: Biomedicines. 2022 Jan 20;10(2):221. doi: 10.3390/biomedicines10020221 (PMC8869621; doi:10.3390/biomedicines10020221)
Supplement: Supplementary file 1 [file biomedicines-10-00221-s001.zip › biomedicines-1494616-supplementary.pdf]

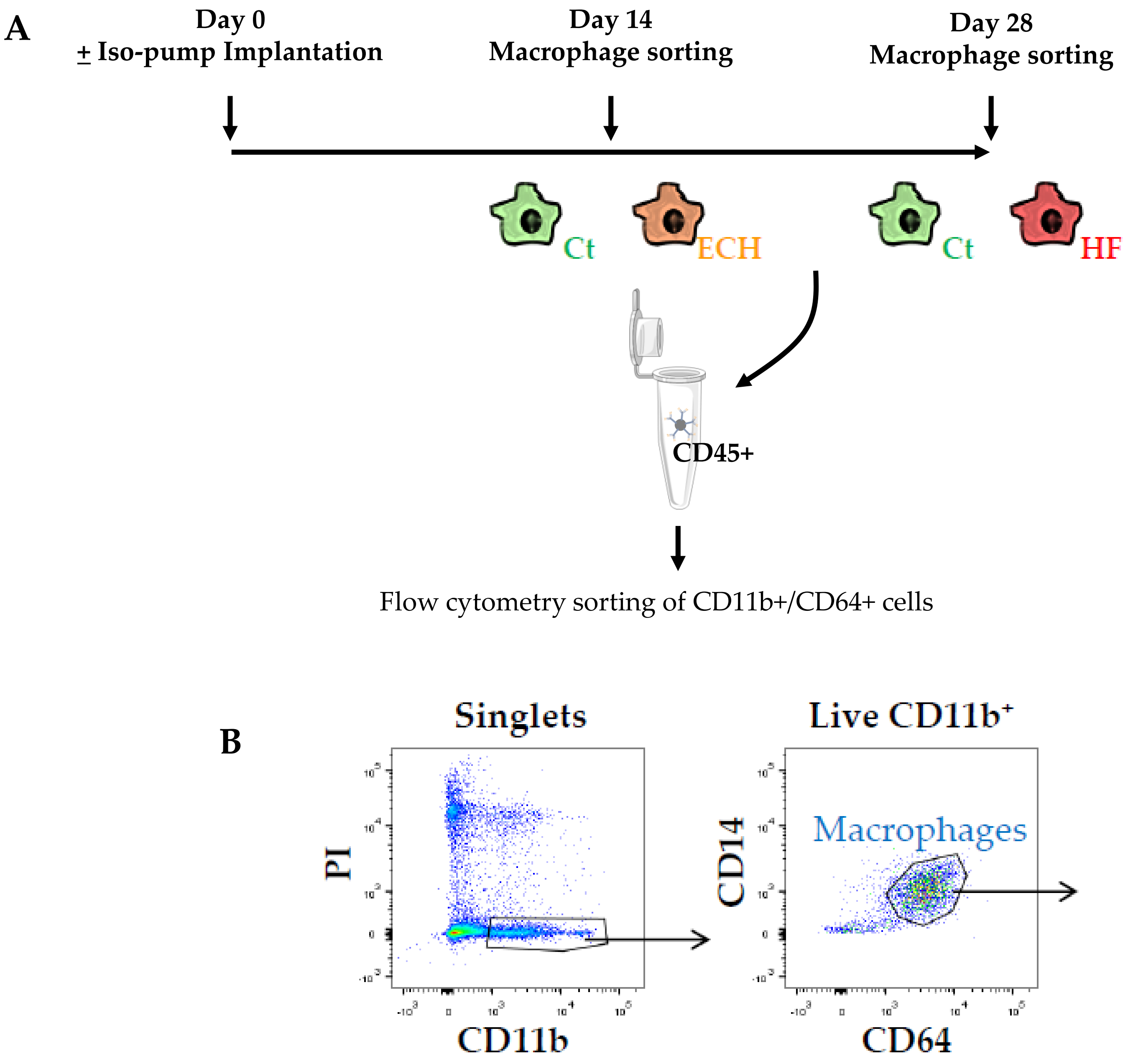


**Figure S1. Time-lapse of macrophage sorting and gating strategy for RNA sequencing and lipidomic analysis. (A)** Schematic representation of iso-pump protocol and time-lapse of immune cells sorting, from collagenase-digested mouse hearts, using mouse CD45 microbeads. **(B)** Flow-cytometry gating strategy to isolate $CD14^+/CD64^+$ macrophages from CD45+ immune cells. Cells were stained with the indicated antibodies, doublets were excluded by FSC-W vs SSCA, and live $CD11^+$ cells were gated on $CD14^+/CD64^+$ after PI exclusion to sort macrophages.

**A** Metascape analysis: enriched ontology clusters
ECH macrophages

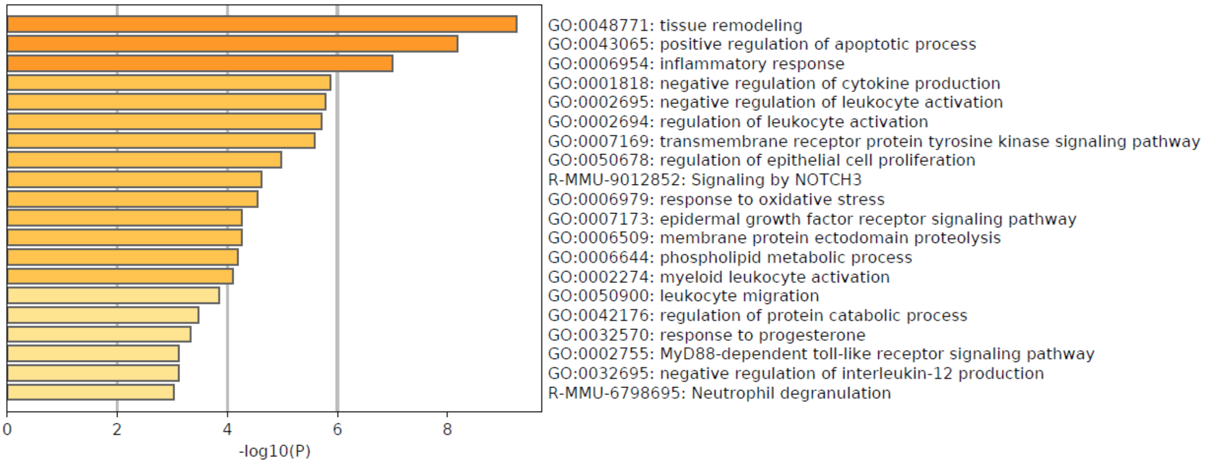


**B** HF macrophages

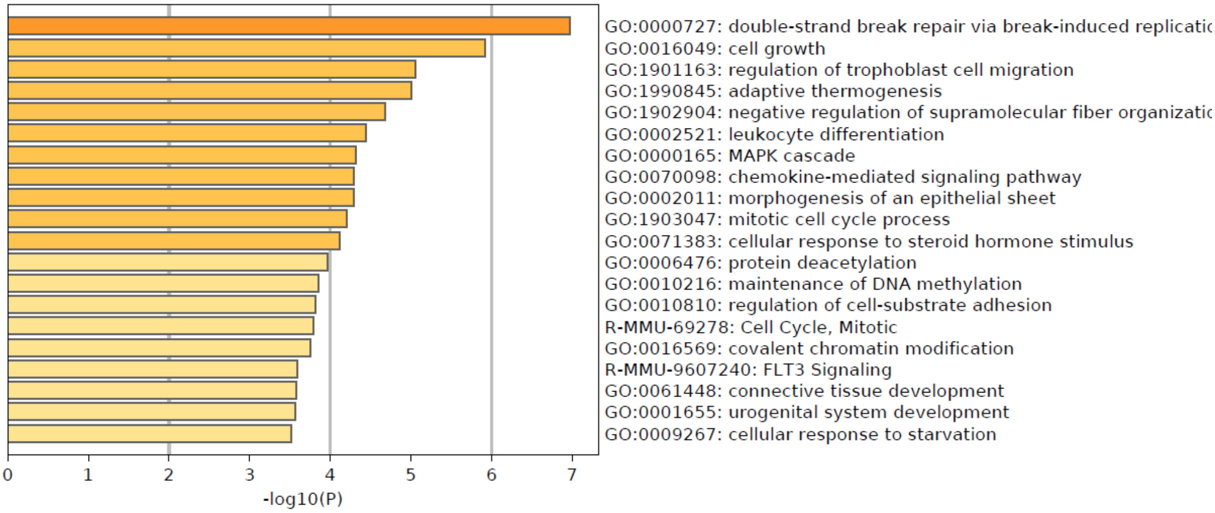


**Table S1: Transcriptomic characterization of ECH and HF cardiac CD64⁺ macrophages.** (A) and (B) Metascape analysis showing specific canonical pathways and functions selectively enriched in ECH and HF macrophages, respectively. Pathways and functions are ranked according to their statistical enrichment in the macrophage transcriptome in ECH or HF conditions. The most highly enriched pathways and functions in ECH (part A) and in HF (part B) macrophages are in dark orange followed by light orange and yellow bars. The *p* value is expressed as –log10.

A

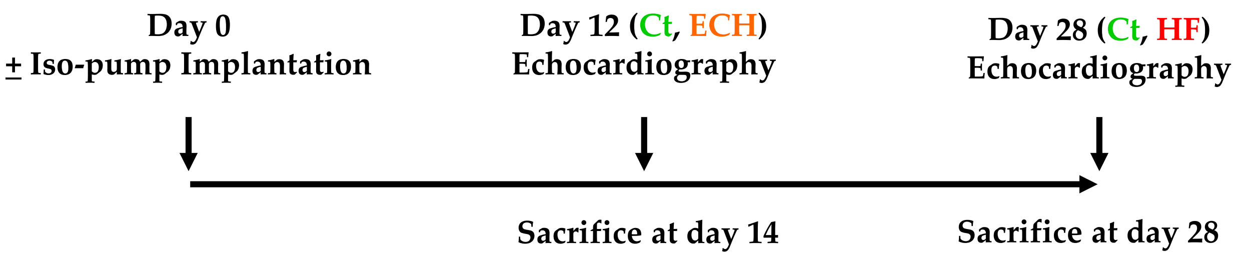


B

| Parameter | Ct (*n* = *11*) | Iso at d12(*n* = *20*) | Iso at d28 (*n* = *6*) |
|---|---|---|---|
| | **Ct** | **ECH** | **HF** |
| **HW/TL** | 7.22 ± 0.2 | 9.12 ± 0.3 * | 8.73 ± 0.4 * |
| **EF (%)** | 83 ± 0.42 | 82 ± 0.8 † | 65 ± 1.5 * |
| **FS (%)** | 46 ± 0.46 | 45 ± 3.7 † | 31 ± 1.1 * |
| **LVd (mm)** | 3.54 ± 0.16 | 3.38 ± 0.11 † | 4.15 ± 0.15 * |
| **LVs (mm)** | 1.9 ± 0.04 | 1.86 ± 0.08 † | 2.86 ± 0.12 * |
| **IVSd (mm)** | 0.62 ± 0.01 | 1 ± 0.02 * | 0.85 ± 0.05 |
| **IVSs (mm)** | 1.11 ± 0.02 | 1.45 ± 0.03 *† | 1.16 ± 0.04 |
| **PWd (mm)** | 0.55 ± 0.03 | 0.85 ± 0.03 * | 0.7 ± 0.04 |
| **PWs (mm)** | 1.04 ± 0.04 | 1.2 ± 0.04 * † | 0.95 ± 0.06 |
| **h/r** | 0.33 ± 0.007 | 0.55 ± 0.02 * † | 0.37 ± 0.03 |
| **HR (bpm)** | 639 ± 5 | 641 ± 13 | 622 ± 6 |

Animals used in lipidomic analysis.

**Figure S2. Mice used in lipidomic analysis. Schematic representation of Iso-pump protocol with time-lapse of echocardiographic and morphologic measurements (A) and echocardiography and morphology parameters (B).**
HR, heart rate; IVSd, end-diastolic interventricular septum thickness; LVd, end-diastolic left ventricular diameter; PWd, end-diastolic posterior wall thickness; IVSs, end-systolic interventricular septum thickness; LVs, end-systolic left ventricular diameter; PWs, end-systolic posterior wall thickness; h/r, diastolic wall thickness to radius ratio; EF, ejection fraction; FS, fractional shortening; HW/TL, heart weight/tibia length.
Kruskal-Wallis followed by Dunn post-hoc tests. * $p<0.05$ ECH or HF vs Ct;
† $p<0.05$ ECH vs HF.
Of note, animals from the HF group (6 mice), prepared in parallel, were not exploited for lipidomic but for morphologic and echographic comparison as well as for statistical analysis.
